# Peritoneal Tuberculosis Mimicking Peritoneal Carcinomatosis in an Immunocompetent Patient

**DOI:** 10.7759/cureus.31464

**Published:** 2022-11-13

**Authors:** Tony Abi El Hessen, Shahzaib Saleem, Riad H Hani, Fatima Z Chadli, Jawad A Makarem

**Affiliations:** 1 Internal Medicine, Dr. Mohamad Amine Zbeib Polyclinic, Doha, QAT; 2 Respiratory/Internal Medicine, Dr. Mohamad Amine Zbeib Polyclinic, Doha, QAT; 3 General Surgery, Al Jabal Hospital, Qornayel, LBN; 4 Radiology, Dr. Mohamad Amine Zbeib Polyclinic, Doha, QAT; 5 Oncology, Al Jabal Hospital, Qornayel, LBN

**Keywords:** ovarian neoplasm, metastatic lymphadenopathy, ascites, peritoneal carcinomatosis, peritoneal tuberculosis (tb)

## Abstract

Peritoneal tuberculosis (TB) is a rare disease among the general population that can be seen in patients with associated immunocompromised conditions such as diabetes mellitus, human immunodeficiency virus (HIV)-positive patients, patients with liver cirrhosis, patients on peritoneal dialysis, and patients on treatment with anti-tumor necrosis factor (TNF) agents. Patients who already have active pulmonary TB and who are not treated promptly can develop disseminated disease within the lungs or can affect extrapulmonary organ systems such as the nervous system, gastrointestinal system, or urinary system. It is unusual to see an otherwise healthy person develop peritoneal TB as a first-time diagnosis, without any previous exposure to TB or any immunocompromising condition.

The diagnosis of this condition can be tricky as the clinical and radiological manifestations of this disease strongly mimic that of malignancy, such as ovarian cancer or peritoneal carcinomatosis. In the majority of cases, the first impression of malignancy is made while examining the radiological images of the abdomen, and only after obtaining the biopsy results, an unexpected diagnosis of peritoneal TB is established. Hence, it is an interesting and uncommon diagnosis, which should always be kept in mind while managing patients with an apparent gynecological malignancy.

Here, we report a case of a 65-year-old female patient who presented with a history of abdominal pain and weight loss. Initial investigation with abdominal ultrasonography revealed ascites with multiple sub-centimeter mesenteric lymphadenopathies. She also had an elevated cancer antigen 125 (CA-125), which further raised suspicion of gynecological malignancy. However, following the investigations, it was found that the actual diagnosis was an unexpected one.

## Introduction

Abdominal tuberculosis (TB) is an umbrella term that can involve any of the abdominal organs such as the gastrointestinal tract, lymph nodes, peritoneum, and solid organs [1]. Of all TB cases around the world, abdominal TB makes up about 5% of the cases [2]. Although a rare disease, it can be diagnosed in patients with immunocompromised status, such as patients receiving treatment with corticosteroids and anti-tumor necrosis factor (TNF) agents, and those with malnutrition, underlying malignancy, diabetes mellitus, human immunodeficiency virus (HIV) infection, and liver cirrhosis [3]. The symptoms of abdominal TB can be variable among patients depending on the organs involved within the abdomen. The most common disease includes the involvement of the peritoneum, intestines, or lymph nodes. Usually, such kinds of patients present with a new onset of ascites, fatigue, fever, diarrhea, abdominal mass, and weight loss [4]. In female patients, specifically, it raises a concern of an intra-abdominal or gynecological malignancy. TB in the abdomen may occur due to the reactivation of latent TB or due to the dissemination of existing TB. It is very rare to see a patient who is otherwise healthy presenting with abdominal TB. Laboratory investigations usually demonstrate an increased level of erythrocyte sedimentation rate (ESR) and anemia [5]. It is also concluded from studies that 15%-25% of patients with abdominal TB have concomitant pulmonary TB [6].

More than 90% of patients presenting with peritoneal TB have ascites at presentation, and the remaining 10% present with more advanced, “dry and doughy” abdomen [7]. Abdominal pain and fever are the other two symptoms that are found in a majority of cases. Peritoneal TB can also occur via hematogenous spread in the setting of active pulmonary TB or miliary TB. Much less commonly, tuberculous mycobacteria enter the peritoneal cavity transmurally from an infected small intestine or via contiguous spread from tuberculous salpingitis [8]. A timely diagnosis of this disease is the key element in improving the outcomes of the patient. In female patients, the diagnosis becomes challenging when the presenting symptoms point toward nothing other than a gynecological malignancy.

## Case presentation

A 65-year-old female patient with a background history of hypertension presented to the emergency department (ED) of Al Jabal Hospital, Lebanon, with symptoms of vague abdominal pain and weight loss of almost 10 kg for a duration of 2-3 months. She had no history of nocturnal fever or cough and no close contact with known TB patients. Physical examination was remarkable for hepatomegaly and ascites. Following this, an abdominal/pelvis ultrasound was organized, which confirmed the findings of the abdominal examination. However, it did not show any evidence of intra-abdominal malignancy. The pelvic organs were also unremarkable. Her chest X-ray was normal. It was then decided to perform computed tomography (CT) of the abdomen and pelvis for her. The CT scan also confirmed hepatomegaly with ascites, but it also revealed multiple sub-centimetric mesenteric lymphadenopathies. The remaining abdominal and pelvic organs such as the spleen, kidneys, gallbladder, ovaries, uterus, fallopian tubes, and urinary bladder were reported as normal.

Her laboratory test results at the time of admission were remarkable for a low hemoglobin level of 9.9 g/dL, a raised cancer antigen 125 (CA-125) of 861 U/mL, and a low albumin level of 29 g/L. Based on these findings, an abdominal ascetic tap was performed, which showed a protein level of 4.6 g/dL, red blood cell (RBC) of 60 cells/mL, white blood cell (WBC) of 320 cells/mL (70% lymphocytes), and a negative reverse transcription-polymerase chain reaction (RT-PCR) for tuberculosis. The fluid did not reveal any acid-fast bacilli on Ziehl-Neelsen staining; however, it was sent for culture.

**Figure 1:**
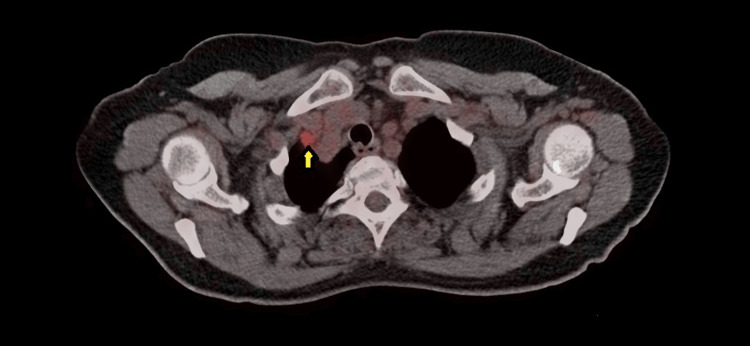
Right supraclavicular lymph node identified on the PET scan (yellow arrow). PET: positron emission tomography

**Figure 2:**
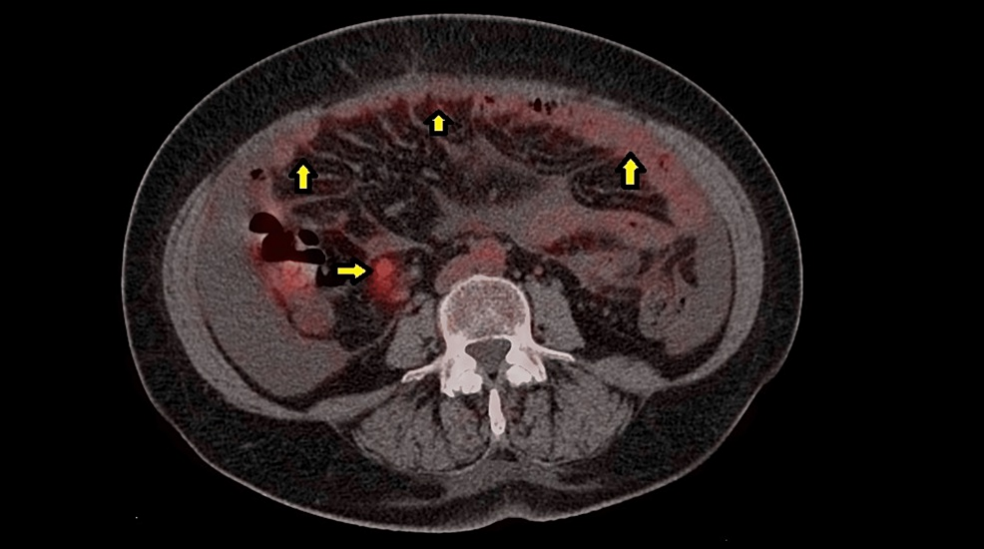
FDG avid lymph nodes and multiple areas of high uptake of the peritoneum (yellow arrows) noted on the PET scan. FDG: fluorodeoxyglucose, PET: positron emission tomography

**Figure 3:**
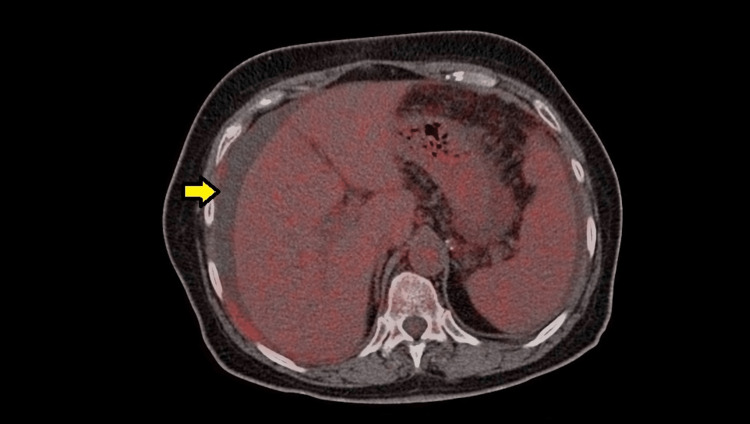
Presence of ascites noted on the PET scan (yellow arrow). PET: positron emission tomography

**Figure 4:**
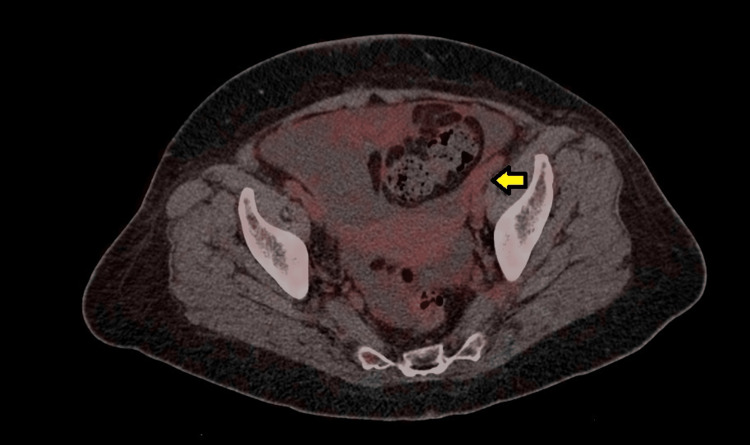
Enlargement of the adnexa seen on the PET scan (yellow arrow). PET: positron emission tomography

At this point, oncology was consulted, and a decision to perform a positron emission tomography (PET) scan was made. The PET scan showed hypermetabolic activity in the right supraclavicular (Figure 1), internal mammary and mesenteric lymph nodes with a maximum standardized uptake value (SUV max) of 2.5, and hypermetabolic activity of the peritoneum with an SUV max of 3.1 (Figure 2). Ascites were noted once again (Figure 3). The scan also showed bilateral adnexal enlargement with mild heterogeneous uptake with an SUV max of 3.0, favoring metastatic primary ovarian neoplasm (Figure 4). Following the PET scan, the patient was readmitted for laparoscopic surgical exploration and biopsies. The samples were successfully obtained from various sites of the omentum and peritoneum. On gross examination during the surgery, the peritoneum was found to have inflammatory nodules, and the bowel loops and ovaries appeared to be normal. Surprisingly, the pathology of these inflammatory nodules was reported as “granulomatous inflammation mostly consistent with tuberculosis. Numerous granulomas are made up of epithelioid and multinucleated giant cells. The granulomas are often surrounded by a fibrotic rim. No malignant cells seen.”

**Figure 5:**
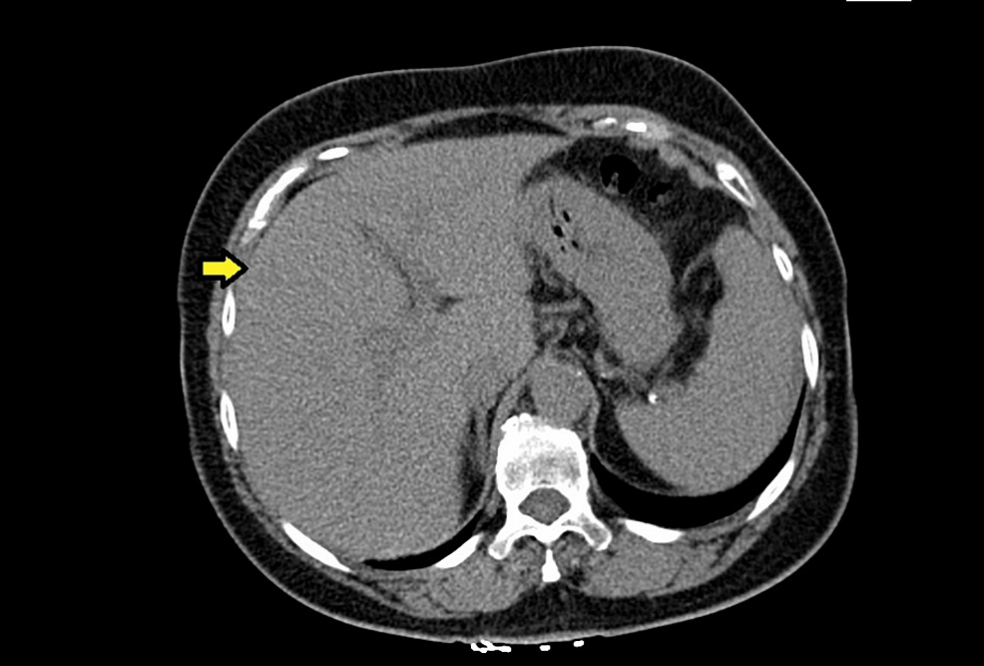
Repeated CT scan showing a complete resolution of previously seen ascites on the PET scan (yellow arrow). CT: computed tomography, PET: positron emission tomography

**Figure 6:**
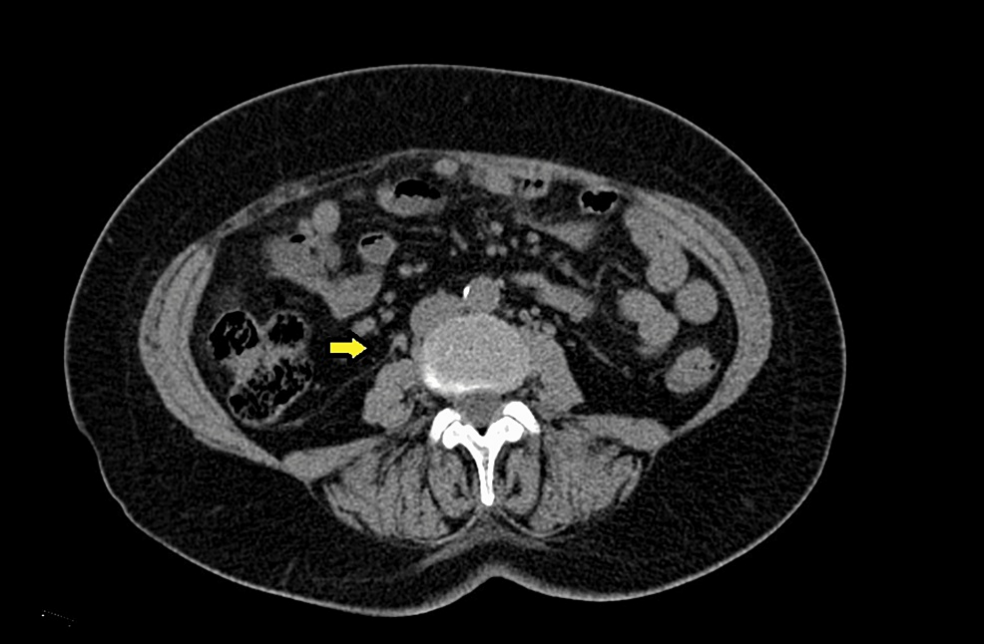
Follow-up CT scan showing smaller and resolving lymphadenopathy (yellow arrow), which were previously noted on the PET scan. CT: computed tomography, PET: positron emission tomography

After the diagnosis of peritoneal TB was confirmed, the patient was immediately commenced on standard antituberculous treatment with isoniazid, rifampicin, ethambutol, and pyrazinamide for a total of six-month duration. During her follow-up, after four months of anti-TB treatment, the patient reported weight gain and resolution of abdominal swelling. After she completed the treatment, another PET scan was arranged, which showed a resolution of ascites (Figure 5) and a resolution of hypermetabolic supraclavicular, internal mammary, and mediastinal lymphadenopathy. The diffuse peritoneal thickening was also resolved. There was some residual activity at two mesenteric nodes noted in the right lower quadrant (Figure 6), and for this reason, the anti-TB treatment was continued for another three months. Following the completion of treatment, she was followed up in the outpatients every three months with a total duration of 12 months. During the follow-up period, she gained weight and did not report any new symptoms related to her treated condition.

## Discussion

This case of peritoneal TB mimicking neoplasm is reported to signify the importance of differential diagnosis when it comes to patients presenting with ascites and weight loss without any history of exposure to TB. Although all investigations performed prior to the biopsies of the lesions were strongly pointing toward a diagnosis of metastatic disease, in reality, that was not the case. It is more common to see peritoneal carcinomatosis mimicking peritoneal TB, as reported by Jung et al. [9], but it is quite rare to see it the other way around. Another important point to discuss here is the importance of CA-125. This tumor marker has been found to be a nonspecific and unhelpful investigation in differentiating the two pathologies. Any kind of peritoneal disease has been associated with an increased level of CA-125, and hence, it is an unreliable investigation in this case. This is also supported by a similar case report by Purbadi et al., where the CA-125 level was found to be 1,200 U/mL [10], which is much higher than our case.

According to a large population-based cohort study, Funston et al. found that the sensitivity and specificity of CA-125 while diagnosing primary ovarian cancer are 77% and 93.8%, respectively. The sensitivity increases to 84.9% in the case of invasive ovarian cancers, whereas the specificity remains the same. The positive predictive value (PPV) and negative predictive value (NPV) for CA-125 were found to be 10.1% and 99.8%, respectively, in cases of ovarian cancers, which signifies the use of CA-125 as a noninvasive screening tool for ovarian cancers. Interestingly, it was found that the sensitivity of CA-125 falls dramatically to 29.1% in cases of non-ovarian cancers. However, the specificity remains around the same value of 94.4% [11].

Multiple factors are reported in the literature that can affect the serum CA-125 results. A comprehensive review by Charkhchi et al. showed that CA-125 can be elevated in a number of benign conditions as well, such as endometriosis, uterine fibroids, inflammation of the peritoneum due to any reason, menstruation, pregnancy, obesity, heart failure, and liver cirrhosis. Abnormal elevations of CA-125 levels are also present in multiple non-ovarian malignancies. For instance, elevated CA-125 levels have been known as a prognostic marker in breast and lung cancers. For postmenopausal women with high CA-125 levels, the presence of lung and breast cancer is recommended to be investigated after the presence of ovarian cancer is excluded. CA-125 is also a useful prognostic marker for pancreatic, colorectal, endometrial, and gastric cancers [12].

Although imaging studies are of utmost importance when it comes to a diagnosis of metastatic disease, biopsy and histopathology are the gold standards. This is proven by our case report, in which all the clinical, biochemical, anatomical, and radiological findings were suggestive of a diagnosis of neoplastic diseases, which turned out to be something totally different once the histological results were obtained. Even the best radiological scans such as PET CT can be deceiving. According to Harkirat et al., PET CT is not a specific investigation for tuberculosis; however, it plays an important role in the evaluation of known or suspected TB cases. PET CT can determine the activity of lesions, guide biopsy from active sites, assess disease extent, detect occult distant foci, and evaluate response to therapy. Active tuberculous lesions often exhibit a high degree of metabolic activity, although this can vary depending on the grade of inflammation. No characteristic pattern has been identified as yet that will definitely differentiate them from cancerous lesions. When PET/CT findings show cancer patients with increased fluorodeoxyglucose (FDG) uptake, tuberculosis should be considered a differential diagnosis, except in suspected cases of metastatic lesions. Tumor markers are not specific indexes in the differential diagnosis of tuberculosis and metastasic diseases [13].

The treatment and follow-up for peritoneal TB are based on the culture and sensitivities of the mycobacterial strain isolated [14]. In the case of fully sensitive organisms, the approach is similar to pulmonary TB. In our case, a six-month regime was offered to the patient, with the first two months of the initial intensive phase with isoniazid, rifampicin, ethambutol, and pyrazinamide, followed by four months of the continuation phase with isoniazid and rifampicin alone. A follow-up CT scan after the completion of treatment should also be performed to evaluate the previously seen lesions and lymphadenopathy. In our case, the follow-up CT scan did show some resolution of lesions and smaller lymph nodes as compared to the previous scan, and this is the reason why the continuation phase of the TB treatment was extended to another three months. However, no more ascites or any new intra-abdominal disease was noted. The patient was then followed up on a regular basis, and she did not report having any abdominal discomfort or swelling. She also gained weight during her follow-up visits. This also signifies the use of CT scans while following up with patients with peritoneal TB as an investigation of choice, and it can also prove to be a reliable investigation in deciding the length of antituberculous treatment.

## Conclusions

Peritoneal TB is a rare diagnosis in an otherwise healthy, immunocompetent patient. This case report highlights the importance of histology when it comes to radiological findings suggesting a diagnosis of malignancy. Peritoneal TB should be considered in any patient presenting with clinical features of abdominal malignancy even in non-endemic regions of the world, and proper attention should be paid to establishing the right diagnosis. Once the treatment is initiated, patients should be followed up with serial CT scans, and this can also be used as a guide toward antituberculous treatment duration.
